# Data on the sub-chronic toxicity in rats after 30 days of oral realgar administration and the accumulation and distribution of arsenic species

**DOI:** 10.1016/j.dib.2018.12.011

**Published:** 2018-12-07

**Authors:** Yan Yi, Shuangrong Gao, Jing Xia, Chunying Li, Yong Zhao, Yushi Zhang, Aihua Liang, Shen Ji

**Affiliations:** a Key Laboratory of Beijing for Identification and Safety Evaluation of Chinese Medicine, Institute of Chinese Materia Medica, China Academy of Chinese Medical Sciences, 16 Nanxiaojie, Dongzhimen Nei, Beijing 100700, China; b Shanghai Institute for Food and Drug Control, No. 1500 Shanghai Zhang Heng Road, Pudong New District, Shanghai 201203, China

## Abstract

These data are related to the research “The accumulation and distribution of arsenic species and association with arsenic toxicity in rats after 30 days of oral realgar administration” (Yi et al., 2019) [1]. These data include the rat body weights, haematology, electrolytes, coagulation and biochemical parameters, and relative organ weights after 30 days of oral administration of realgar, which was consistent with the current OECD guideline “Repeated Dose 28-Day oral Toxicity Study in Rodents”. The data also include the content of arsenite (As(III)), arsenate (As(V)), dimethylarsinic acid (DMA), monomethylarsonic acid (MMAV), arsenic betaine (AsB) and arsenic chrome (AsC) in rat blood, liver, kidneys, brain and urine after single-dose and 30-day oral administration of realgar.

The provided data are intended to demonstrate whether realgar has short-term toxicity and the role of accumulated arsenic species in realgar toxicity.

**Specifications table**

**Table:** 

Subject area	Toxicology
More specific subject area	General toxicity effect
Type of data	Tables, figures
How data were acquired	Rat toxicity test data were collected according to the M3 guidelines developed by ICH for non-clinical repeated dose toxicity. Measurements of arsenic metabolites and accumulation were performed on an Agilent 7700ce ICP-MS instrument coupled with the Agilent 1200 Series HPLC system.
Data format	Raw and analysed
Experimental factors	To analyze the short-term toxicity of realgar, rats were treated with different doses of realgar for 30 days. Then, the function and tissue structure changes of the whole-body organs of the rats were tested. Analysis of total arsenic (tAs) and arsenic (As) in rat blood and organs using the Agilent 7700ce ICP-MS instrument and the Agilent 1200 Series HPLC system.
Experimental features	Studies were performed under GLP conditions according the current OECD Test guideline TG407 “Repeated Dose 28-Day oral Toxicity Study in Rodents”. Comparison of various arsenic species accumulation and distribution.
Data source location	Institute of Chinese Materia Medica, China Academy of Chinese Medical Sciences, Beijing, China
Data accessibility	Data available within the article.
Related research article	Yi, Y., Gao, S., Xia, J., Li, C., Zhao, Y., Zhang, Y., Liang A., Ji S. Study of the accumulation and distribution of arsenic species and association with arsenic toxicity in rats after 30 days of oral realgar administration. Journal of Ethnopharmacology. 2019, 11576 [1].

**Value of the data**

• The data show the repeated-dose 30-day oral toxicity of realgar in rats, which provides a reference for the safe dose range for repeated oral administration of realgar for 2 weeks in humans.
• The accumulation and distribution of arsenic species in rat tissues not only helps in understanding the metabolism of arsenic in animals but also helps elucidate the main arsenic-accumulating organs in the body.
• The total arsenic content is not sufficient for the safety evaluation of realgar. The data revealed a possible link between arsenic species and the sub-chronic toxicity of realgar, which can help people study the toxicity of realgar more scientifically and find reasonable prevention and treatment.

## 1 Data

The content of arsenic species in rats after a single administration of realgar is shown in Table 1, Table 2, Table 3, Table 4, Table 5. The body weights (Table 6, Table 7, Table 8), haematology, electrolytes, coagulation (Table 9, Table 10), biochemical parameters (Table 11, Table 12) and relative organ weights (Table 13, Table 14) of male and female rats after 30 days of administration of realgar are shown below. In addition, the content of arsenic species in the rats is shown in Table 15, Table 16, Table 17.

**Table 1:** Content of arsenic species in the livers of rats from 0 h to 48 h after a single administration of realgar (n = 6, 3 males and 3 females per group, $\bar{x}$ ± SD).

Time	AsC	AsB	As(III)	DMA	MMA	As(V)	tAs
0 h	0.00 ± 0.00	0.03 ± 0.01	0.00 ± 0.00	0.28 ± 0.04	0.02 ± 0.02	0.03 ± 0.05	0.82 ± 0.29
0.25 h	0.00 ± 0.00	0.04 ± 0.01	2.54 ± 1.33^*^	0.67 ± 0.38	0.34 ± 0.14	0.12 ± 0.04^**^	5.16 ± 1.91^**^
0.5 h	0.00 ± 0.00	0.02 ± 0.01	4.03 ± 1.91^**^	0.63 ± 0.26^*^	0.49 ± 0.12^*^	0.27 ± 0.11^***^	7.55 ± 1.82^**^
1 h	0.00 ± 0.00	0.02 ± 0.01	2.58 ± 0.98^**^	0.91 ± 0.99	0.49 ± 0.14^**^	0.16 ± 0.04^***^	6.72 ± 2.34^**^
2 h	0.00 ± 0.00	0.02 ± 0.01	2.62 ± 1.48^**^	1.51 ± 0.36^**^	0.44 ± 0.23^*^	0.12 ± 0.08^*^	6.98 ± 2.79^**^
4 h	0.00 ± 0.00	0.03 ± 0.01	0.89 ± 0.31^**^	1.52 ± 0.17^**^	0.17 ± 0.06	0.10 ± 0.05^*^	5.18 ± 0.71^**^
8 h	0.00 ± 0.00	0.02 ± 0.01	0.30 ± 0.04^**^	1.86 ± 0.17^*^	0.06 ± 0.06	0.05 ± 0.05	4.14 ± 0.73^**^
16 h	0.00 ± 0.00	0.02 ± 0.01	0.20 ± 0.04^*^	2.58 ± 0.17^**^	0.03 ± 0.03	0.03 ± 0.04	4.30 ± 0.82^**^
32 h	0.00 ± 0.00	0.04 ± 0.02	0.07 ± 0.04^*^	1.74 ± 0.28^**^	0.01 ± 0.02	0.03 ± 0.03	2.93 ± 0.73^*^
48 h	0.00 ± 0.00	0.03 ± 0.01	0.06 ± 0.04^*^	1.39 ± 0.89^**^	0.02 ± 0.01	0.04 ± 0.06	2.09 ± 1.17^*^

All values are expressed as the means (M) ± standard deviation (SD). ^*^p <0.05, ^**^p < 0.01, ^***^p < 0.001. Values are compared with the content at 0 h. Differences were analysed by a one-way analysis of variance.

**Table 2:** Content of arsenic species in the kidneys of rats from 0 h to 48 h after a single administration of realgar (*n* = 6, 3 males and 3 females per group, $\bar{x}$ ± SD).

Time	AsC	AsB	As(III)	DMA	MMA	As(V)	tAs
0 h	0.00 ± 0.00	0.00 ± 0.00	0.07 ± 0.02	0.11 ± 0.05	0.00 ± 0.00	0.19 ± 0.07	0.38 ± 0.28
0.25 h	0.00 ± 0.00	0.00 ± 0.00	1.32 ± 0.66^*^	0.22 ± 0.05^*^	0.19 ± 0.11^*^	1.72 ± 0.76^**^	2.50 ± 1.31^**^
0.5 h	0.00 ± 0.00	0.00 ± 0.00	3.32 ± 1.41^**^	0.24 ± 0.06^*^	0.38 ± 0.08^**^	3.98 ± 1.39^***^	6.77 ± 2.86^***^
1 h	0.00 ± 0.00	0.00 ± 0.00	1.60 ± 1.13^**^	0.29 ± 0.12^*^	0.40 ± 0.26^**^	2.32 ± 1.45^**^	3.64 ± 1.94^**^
2 h	0.00 ± 0.00	0.00 ± 0.00	2.46 ± 1.12	0.54 ± 0.07^**^	0.74 ± 0.30^**^	3.76 ± 1.34^***^	8.61 ± 5.01^***^
4 h	0.00 ± 0.00	0.00 ± 0.00	0.53 ± 0.27^**^	0.60 ± 0.10^**^	0.31 ± 0.13^**^	1.45 ± 0.47^**^	2.27 ± 0.69^**^
8 h	0.00 ± 0.00	0.00 ± 0.00	0.18 ± 0.08^**^	0.57 ± 0.06^**^	0.16 ± 0.05^**^	0.92 ± 0.12^**^	1.57 ± 0.33^**^
16 h	0.00 ± 0.00	0.00 ± 0.00	0.15 ± 0.03^**^	0.54 ± 0.12^**^	0.19 ± 0.12^*^	0.87 ± 0.25^**^	1.78 ± 0.76^**^
32 h	0.00 ± 0.00	0.00 ± 0.00	0.12 ± 0.04^*^	0.69 ± 0.13^**^	0.07 ± 0.08^*^	0.88 ± 0.22^**^	1.63 ± 0.75^**^
48 h	0.00 ± 0.00	0.00 ± 0.00	0.13 ± 0.02^*^	0.82 ± 0.26^**^	0.21 ± 0.15^*^	1.16 ± 0.24^**^	2.03 ± 0.27^***^

All values are expressed as the means (M) ± standard deviation (SD). ^*^p < 0.05, ^**^p < 0.01, ^***^p < 0.001. Values are compared with the content at 0 h. Differences were analysed by a one-way analysis of variance.

**Table 3:** Content of arsenic species in the brains of rats from 0 h to 48 h after a single administration of realgar (*n* = 6, 3 males and 3 females per group, $\bar{x}$ ± SD).

Time	AsC	AsB	As(III)	DMA	MMA	As(V)	tAs
0 h	0.00 ± 0.00	0.00 ± 0.00	0.04 ± 0.02	0.06 ± 0.02	0.00 ± 0.00	0.56 ± 0.09	1.35 ± 0.30
0.25 h	0.00 ± 0.00	0.00 ± 0.00	0.06 ± 0.02	0.08 ± 0.03	0.00 ± 0.00	0.64 ± 0.14	2.00 ± 0.29^**^
0.5 h	0.00 ± 0.00	0.00 ± 0.00	0.08 ± 0.03	0.09 ± 0.03	0.00 ± 0.00	0.56 ± 0.07	1.71 ± 0.20
1 h	0.00 ± 0.00	0.00 ± 0.00	0.07 ± 0.02	0.11 ± 0.03	0.00 ± 0.00	0.59 ± 0.06	1.90 ± 0.28^*^
2 h	0.00 ± 0.00	0.00 ± 0.00	0.09 ± 0.05	0.19 ± 0.06	0.00 ± 0.00	0.57 ± 0.07	1.81 ± 0.21^*^
4 h	0.00 ± 0.00	0.00 ± 0.00	0.09 ± 0.05	0.31 ± 0.07^**^	0.00 ± 0.00	0.56 ± 0.05	1.77 ± 0.30
8 h	0.00 ± 0.00	0.00 ± 0.00	0.05 ± 0.01	0.44 ± 0.06^***^	0.00 ± 0.00	0.56 ± 0.06	2.01 ± 0.46^**^
16 h	0.00 ± 0.00	0.00 ± 0.00	0.06 ± 0.01	0.43 ± 0.13^**^	0.00 ± 0.00	0.58 ± 0.05	1.87 ± 0.37^*^
32 h	0.00 ± 0.00	0.00 ± 0.00	0.05 ± 0.01	0.43 ± 0.06^***^	0.00 ± 0.00	0.57 ± 0.09	2.17 ± 0.27^**^
48 h	0.00 ± 0.00	0.00 ± 0.00	0.06 ± 0.02	0.40 ± 0.04^***^	0.00 ± 0.00	0.59 ± 0.10	2.05 ± 0.51^**^

All values are expressed as the means (M) ± standard deviation (SD). ^*^p < 0.05, ^**^p < 0.01, ^***^p < 0.001. Values are compared with the content at 0 h. Differences were analysed by a one-way analysis of variance.

**Table 4:** Content of arsenic species in the plasma of rats from 0 h to 48 h after a single administration of realgar (*n* = 6, 3 males and 3 females per group, $\bar{x}$ ± SD).

Time	AsC	AsB	As(III)	DMA	MMA	As(V)	tAs
0 h	0.00 ± 0.00	0.00 ± 0.00	0.00 ± 0.00	3.88 ± 0.06	0.00 ± 0.00	0.19 ± 0.03	4.06 ± 0.70
0.25 h	0.00 ± 0.00	0.00 ± 0.00	0.00 ± 0.00	3.53 ± 0.27	0.00 ± 0.00	0.22 ± 0.02	3.73 ± 0.35
0.5 h	0.00 ± 0.00	0.00 ± 0.00	0.00 ± 0.00	3.94 ± 1.46	0.00 ± 0.00	0.24 ± 0.04	4.24 ± 1.51
1 h	0.00 ± 0.00	0.00 ± 0.00	0.00 ± 0.00	4.85 ± 1.23	0.00 ± 0.00	0.23 ± 0.04	5.20 ± 0.19
2 h	0.00 ± 0.00	0.00 ± 0.00	0.00 ± 0.00	6.96 ± 2.41^*^	0.00 ± 0.00	0.23 ± 0.02	7.29 ± 2.42^*^
4 h	0.00 ± 0.00	0.00 ± 0.00	0.00 ± 0.00	11.85 ± 4.13^**^	0.00 ± 0.00	0.19 ± 0.06	12.40 ± 4.29^**^
8 h	0.00 ± 0.00	0.00 ± 0.00	0.00 ± 0.00	18.80 ± 4.50^***^	0.00 ± 0.00	0.19 ± 0.02	19.05 ± 4.28^***^
16 h	0.00 ± 0.00	0.00 ± 0.00	0.00 ± 0.00	31.75 ± 4.33^***^	0.00 ± 0.00	0.13 ± 0.09	31.60 ± 4.25^***^
32 h	0.00 ± 0.00	0.00 ± 0.00	0.00 ± 0.00	26.95 ± 0.61^***^	0.00 ± 0.00	0.18 ± 0.02	27.10 ± 4.87^***^
48 h	0.00 ± 0.00	0.00 ± 0.00	0.00 ± 0.00	35.70 ± 5.11^***^	0.00 ± 0.00	0.20 ± 0.02	35.95 ± 5.14^***^

All values are expressed as the means (M) ± standard deviation (SD). ^*^p < 0.05, ^**^p < 0.01, ^***^p < 0.001. Values are compared with the content at 0 h. Differences were analysed by a one-way analysis of variance.

**Table 5:** Content of arsenic species in the urine of rats from 0 h to 48 h after a single administration of realgar (*n* = 6, 3 males and 3 females per group, $\bar{x}$ ± SD).

Arsenic species	Control	0–12 h	12–24 h
AsC	0.00 ± 0.00	0.00 ± 0.00	0.00 ± 0.00
AsB	0.00 ± 0.00	0.01 ± 0.00	0.01 ± 0.01
As(III)	0.00 ± 0.00	0.47 ± 0.14^*^	0.09 ± 0.10^*^
DMA	0.00 ± 0.00	3.19 ± 0.18^***^	1.01 ± 0.59^*^
MMA	0.00 ± 0.00	0.71 ± 0.06^**^	0.28 ± 0.28
As(V)	0.00 ± 0.00	0.42 ± 0.02	0.11 ± 0.11
t-As	0.00 ± 0.00	10.30 ± 0.33^***^	3.48 ± 0.51^***^

All values are expressed as the means (M) ± standard deviation (SD). ^*^p < 0.05, ^**^p < 0.01, ^***^p < 0.001. Values are compared with the content at 0 h. Differences were analysed by a one-way analysis of variance.

**Table 6:** Body weights of male rats after treatment with realgar at 170 mg/kg for 30 days (*n* = 5, $\bar{x}$ ± SD).

Time	Control	Realgar		
		10.6 mg/kg	40.5 mg/kg	170 mg/kg
0 d	236.15 ± 11.01	236.00 ± 11.77	234.64 ± 11.65	235.48 ± 13.34
8 d	280.64 ± 16.24	284.06 ± 13.76	281.99 ± 14.99	284.11 ± 15.12
15 d	326.56 ± 23.67	331.36 ± 18.18	328.18 ± 17.69	329.03 ± 19.82
22 d	346.47 ± 28.63	351.55 ± 21.62	349.32 ± 20.06	345.97 ± 22.00
29 d	390.89 ± 32.97	385.82 ± 22.96	387.12 ± 28.14	379.35 ± 35.60

All values are expressed as the means (M) ± standard deviation (SD). ^*^p < 0.05, ^**^p < 0.01, ^***^p < 0.001. The body weights per time are compared to those of the control group. Differences were analysed by a one-way analysis of variance.

**Table 7:** Body weights of female rats after treatment with realgar at 170 mg/kg for 30 days (*n* = 5, $\bar{x}$ ± SD).

Time	Control	Realgar		
		10.6 mg/kg	40.5 mg/kg	170 mg/kg
0 d	182.64 ± 10.40	181.67 ± 12.32	182.65 ± 10.50	182.75 ± 10.20
8 d	204.94 ± 10.62	203.50 ± 13.53	202.33 ± 10.66	205.83 ± 12.78
15 d	218.84 ± 12.35	222.83 ± 18.72	221.51 ± 11.78	223.68 ± 13.99
22 d	231.82 ± 13.84	230.12 ± 20.79	229.27 ± 13.44	237.42 ± 13.88
29 d	248.26 ± 16.50	240.21 ± 30.77	242.82 ± 22.68	246.71 ± 20.75

All values are expressed as the means (M) ± standard deviation (SD). ^*^p < 0.05, ^**^p < 0.01, ^***^p < 0.001. The body weights per time are compared to those of the control group. Differences were analysed by a one-way analysis of variance.

**Table 8:** Haematological data from the oral administration of realgar for 30 days in male rats (*n* = 5, $\bar{x}$ ± SD).

Item	Control	Realgar		
		10.6 mg/kg	40.5 mg/kg	170 mg/kg
**WBC (10**^**9**^**/L)**	4.09 ± 0.45	5.60 ± 1.36	5.49 ± 1.43^*^	5.20 ± 1.15^*^
**RBC (10**^**12**^**/L)**	7.09 ± 0.32	7.05 ± 0.51	7.52 ± 0.25^*^	7.53 ± 0.42
**HGB (g/L)**	127.00 ± 5.48	128.20 ± 6.53	130.60 ± 3.78	135.40 ± 7.64^*^
**HCT (%)**	39.72 ± 1.47	39.52 ± 2.07	40.20 ± 1.20	41.72 ± 2.13
**MCV (fL)**	56.02 ± 1.44	56.12 ± 1.46	53.48 ± 1.22^**^	55.44 ± 0.35
**MCH (pg)**	17.88 ± 0.50	18.24 ± 0.47	17.36 ± 0.40	17.96 ± 0.15
**MCHC (g/L)**	319.20 ± 5.31	324.80 ± 4.55	324.60 ± 2.07^*^	324.40 ± 3.05^*^
**PLT (10**^**9**^**/L)**	1202.20 ± 112.52	1251.40 ± 178.27	1323.00 ± 119.52	1192.60 ± 111.39
**Gr (%)**	70.80 ± 5.85	78.82 ± 4.84^*^	82.90 ± 1.80^**^	79.20 ± 3.12^*^
**Ly (%)**	4.58 ± 0.91	3.02 ± 0.739	2.32 ± 0.56	2.66 ± 0.69
**MO (%)**	24.62 ± 5.42	18.16 ± 4.59^*^	14.44 ± 1.71^**^	17.78 ± 2.69^*^
**PT (S)**	12.20 ± 0.58	12.58 ± 0.42	12.68 ± 0.40	10.98 ± 4.15
**APTT (S)**	19.20 ± 1.31	17.60 ± 1.36^*^	20.32 ± 1.58	18.26 ± 3.15

All values are expressed as the means (M) ± standard deviation (SD). ^*^p < 0.05, ^**^p < 0.01, ^***^p < 0.001. Values are compared with those of the control group. Differences were analysed by a one-way analysis of variance. WBC: white blood cells; RBC: red blood cells; HGB: haemoglobin; HCT: haematocrit; MCV: mean corpuscular volume; MCH: mean corpuscular haemoglobin; MCHC: mean corpuscular haemoglobin concentration; PLT: blood platelet number; GR%: neutrophilic granulocyte ratio; LY%: lymphocyte ratio; MO: monocytes ratio; PT: prothrombin time; APTT: activated partial thromboplastin time.

**Table 9:** Haematological data from the oral administration of realgar for 30 days in female rats (*n* = 5, $\bar{x}$ ± SD).

Item	Control	Realgar		
		10.6 mg/kg	40.5 mg/kg	170 mg/kg
**WBC (10**^**9**^**/L)**	2.51 ± 0.35	3.75 ± 1.35	3.40 ± 0.97^*^	3.17 ± 0.96
**RBC (10**^**12**^**/L)**	6.47 ± 0.29	6.71 ± 0.27	6.66 ± 0.20	6.82 ± 0.19^*^
**HGB (g/L)**	114.00 ± 4.30	119.80 ± 4.49^*^	120.60 ± 3.71^*^	120.80 ± 3.11^*^
**HCT (%)**	35.62 ± 1.01	36.62 ± 1.33	36.52 ± 1.05	36.72 ± 0.59^*^
**MCV (fL)**	55.10 ± 1.01	54.56 ± 1.68	54.86 ± 1.53	53.90 ± 1.59
**MCH (pg)**	17.66 ± 0.51	17.84 ± 0.61	18.14 ± 0.56	17.76 ± 0.44
**MCHC (g/L)**	320.00 ± 6.36	326.80 ± 1.48^*^	330.40 ± 2.07^**^	329.20 ± 4.82^*^
**PLT (10**^**9**^**/L)**	1111.80 ± 119.29	1041.40 ± 86.51	1128.00 ± 41.84	1218.60 ± 85.36
**Gr (%)**	76.42 ± 4.74	80.40 ± 7.44	81.12 ± 5.19	79.00 ± 7.39
**Ly (%)**	3.04 ± 0.98	5.62 ± 6.88	3.46 ± 0.95	3.84 ± 1.00
**MO (%)**	20.04 ± 4.62	13.58 ± 8.14	15.06 ± 5.50	16.72 ± 6.70
**PT (S)**	13.16 ± 0.51	13.62 ± 0.30	13.48 ± 0.41	11.43 ± 4.75
**APTT (S)**	21.62 ± 2.04	21.64 ± 3.76	19.62 ± 2.29	18.96 ± 2.25^*^

All values are expressed as the means (M) ± standard deviation (SD). ^*^p < 0.05, ^**^p < 0.01, ^***^p < 0.001. Values are compared with those of the control group. Differences were analysed by a one-way analysis of variance. WBC: white blood cells; RBC: red blood cells; HGB: haemoglobin; HCT: haematocrit; MCV: mean corpuscular volume; MCH: mean corpuscular haemoglobin; MCHC: mean corpuscular haemoglobin concentration; PLT: blood platelet number; GR%: neutrophilic granulocyte ratio; LY%: lymphocyte ratio; MO: monocytes ratio; PT: prothrombin time; APTT: activated partial thromboplastin time.

**Table 10:** Serum biochemistry data from the oral administration of realgar for 30 days in male rats (*n* = 5, $\bar{x}$ ± SD).

Item	Control	Realgar		
		10.6 mg/kg	40.5 mg/kg	170 mg/kg
**ALB (g/L)**	32.74 ± 1.28	32.30 ± 3.47	31.52 ± 0.74	32.12 ± 1.38
**ALP (IU)**	118.80 ± 16.10	105.20 ± 13.26	110.00 ± 10.27	94.60 ± 5.18^*^
**ALT (U/L)**	36.60 ± 4.98	33.40 ± 4.83	30.60 ± 3.13^*^	31.60 ± 5.86
**AST (U/L)**	113.20 ± 23.13	137.80 ± 20.86	94.00 ± 7.35	128.00 ± 10.56
**T-CHO (mmol/L)**	1.91 ± 0.18	32.30 ± 3.47	31.52 ± 0.74	32.12 ± 1.38
**CK-NAC (U/L)**	378.00 ± 168.30	181.00 ± 120.66^*^	565.20 ± 98.01^*^	379.80 ± 46.24
**CRE (µmol/L)**	45.60 ± 1.67	44.20 ± 1.30	47.80 ± 1.64^*^	45.60 ± 2.51
**γ-GT (U/L)**	0.34 ± 0.18	0.22 ± 0.18	0.20 ± 0.14	0.24 ± 0.09
**GLU (mmol/L)**	5.89 ± 0.18	7.84 ± 0.52^***^	7.88 ± 0.49^***^	7.39 ± 0.25^***^
**BUN (mmol/L)**	5.92 ± 0.26	5.00 ± 0.48^**^	5.14 ± 0.38^**^	5.92 ± 0.70
**TBIL (µmol/L)**	4.48 ± 0.89	4.70 ± 0.65	7.88 ± 3.06^*^	5.00 ± 0.75
**TG (mmol/L)**	0.46 ± 0.11	0.38 ± 0.09	0.37 ± 0.05	0.39 ± 0.18
**TP (g/L)**	55.28 ± 2.70	52.46 ± 0.96^*^	54.24 ± 3.93	52.26 ± 2.47
**Na (mmol/L)**	143.32 ± 5.72	140.30 ± 1.38	138.90 ± 0.84	140.18 ± 1.38
**K (mmol/L)**	4.44 ± 0.43	3.93 ± 0.40^*^	5.02 ± 0.52^*^	4.38 ± 0.16
**Cl (mmol/L)**	109.16 ± 4.67	108.06 ± 1.78	108.56 ± 0.39	108.30 ± 1.41

All values are expressed as the means (M) ± standard deviation (SD). ^*^p < 0.05, ^**^p < 0.01, ^***^p < 0.001. Values are compared with those of the control group. Differences were analysed by a one-way analysis of variance. ALB: total albumin; ALP: alkaline phosphatase; ALT: alanine aminotransferase; AST: aspartate aminotransferase; CHO: cholesterol; CK: creatine kinase; CRE: creatinine; GGT: γ -glutamyl transpeptidase; GLU: glucose; TBIL: total bilirubin; TG: triglycerides; TP: total protein; BUN: blood urea nitrogen; Na+: sodium; K+: potassium; Cl-: chloride.

**Table 11:** Serum biochemistry data from the oral administration of realgar for 30 days in female rats (*n* = 5, $\bar{x}$ ± SD).

Item	Control	Realgar		
		10.6 mg/kg	40.5 mg/kg	170 mg/kg
**ALB (g/L)**	32.24 ± 2.38	29.30 ± 0.53^*^	29.06 ± 1.25^*^	30.34 ± 1.17
**ALP (IU)**	54.40 ± 10.38	72.40 ± 18.72^**^	63.80 ± 14.02	60.80 ± 14.08
**ALT (U/L)**	33.20 ± 6.30	36.00 ± 4.42	25.00 ± 3.08^*^	25.80 ± 3.77^*^
**AST (U/L)**	118.20 ± 13.03	111.60 ± 13.67	113.20 ± 13.88	112.20 ± 30.44
**T-CHO (mmol/L)**	1.42 ± 0.31	1.19 ± 0.18	1.21 ± 0.21	1.30 ± 0.16
**CK-NAC (U/L)**	348.00 ± 135.51	318.60 ± 87.27	371.40 ± 55.56	457.00 ± 239.78
**CRE (µmol/L)**	51.80 ± 2.28	48.00 ± 4.85^**^	47.00 ± 4.64^*^	49.20 ± 5.22
**γ-GT (U/L)**	0.46 ± 0.17	0.46 ± 0.09	0.44 ± 0.25	0.60 ± 0.10
**GLU (mmol/L)**	6.46 ± 0.49	6.65 ± 0.38	6.62 ± 0.48	6.15 ± 0.51
**BUN (mmol/L)**	7.05 ± 1.02	7.55 ± 2.07	5.44 ± 0.58^**^	6.17 ± 1.54
**TBIL (µmol/L)**	3.14 ± 0.32	3.32 ± 0.70	2.70 ± 0.50	3.32 ± 0.67
**TG (mmol/L)**	0.25 ± 0.03	0.27 ± 0.06	0.24 ± 0.03	0.32 ± 0.03^**^
**TP (g/L)**	52.32 ± 2.80	48.38 ± 0.91	48.24 ± 1.51^*^	50.12 ± 2.10
**Na (mmol/L)**	140.72 ± 1.41	141.30 ± 0.74	138.34 ± 3.57	139.72 ± 1.30
**K (mmol/L)**	3.72 ± 0.29	4.23 ± 0.52^*^	3.79 ± 0.18	3.74 ± 0.11
**Cl (mmol/L)**	109.90 ± 1.88	110.82 ± 1.47	108.14 ± 2.88	109.32 ± 0.79

All values are expressed as the means (M) ± standard deviation (SD). ^*^p < 0.05, ^**^p < 0.01, ^***^p < 0.001. Values are compared with those of the control group. Differences were analysed by a one-way analysis of variance. ALB: total albumin; ALP: alkaline phosphatase; ALT: alanine aminotransferase; AST: aspartate aminotransferase; CHO: cholesterol; CK: creatine kinase; CRE: creatinine; GGT: γ -glutamyl transpeptidase; GLU: glucose; TBIL: total bilirubin; TG: triglycerides; TP: total protein; BUN: blood urea nitrogen; Na+: sodium; K+: potassium; Cl-: chloride.

**Table 12:** Relative organ weights data from the oral administration of realgar for 30 days in male rats (%).

Item	Control	Realgar		
		10.6 mg/kg	40.5 mg/kg	170 mg/kg
**Heart**	0.323 ± 0.033	0.334 ± 0.020	0.311 ± 0.022	0.317 ± 0.020
**Liver**	2.952 ± 0.247	3.012 ± 0.120	3.082 ± 0.198	2.514 ± 0.860
**Spleen**	0.250 ± 0.030	0.230 ± 0.167	0.235 ± 0.016	0.269 ± 0.037
**Lung**	0.415 ± 0.046	0.429 ± 0.043	0.433 ± 0.033	0.435 ± 0.028
**Kidney**	0.746 ± 0.034	0.686 ± 0.164	0.728 ± 0.035	0.730 ± 0.061
**Brain**	0.522 ± 0.034	0.560 ± 0.044	0.544 ± 0.026	0.551 ± 0.058
**Stomach**	0.018 ± 0.003	0.020 ± 0.003	0.020 ± 0.003	0.018 ± 0.004
**Adrenal gland**	0.376 ± 0.120	0.401 ± 0.092	0.352 ± 0.085	0.389 ± 0.099
**Thymus**	0.944 ± 0.067	0.778 ± 0.435	0.902 ± 0.144	1.035 ± 0.058
**Testis**	0.256 ± 0.086	0.211 ± 0.035	0.194 ± 0.017^*^	0.230 ± 0.048
**Epididymis**	0.205 ± 0.056	0.182 ± 0.020	0.170 ± 0.074	0.162 ± 0.060

All values are expressed as the means (M) ± standard deviation (SD). ^*^p < 0.05, ^**^p < 0.01, ^***^p < 0.001. Values are compared with those of the control group. Differences were analysed by a one-way analysis of variance.

**Table 13:** Relative organ weights data from the oral administration of realgar for 30 days in female rats (%).

Item	Control	Realgar		
		10.6 mg/kg	40.5 mg/kg	170 mg/kg
**Heart**	0.355 ± 0.016	0.369 ± 0.048	0.370 ± 0.038	0.371 ± 0.026
**Liver**	3.083 ± 0.106	3.124 ± 0.132	2.774 ± 0.073^**^	2.798 ± 0.108^**^
**Spleen**	0.314 ± 0.027	0.275 ± 0.014^*^	0.285 ± 0.015^*^	0.323 ± 0.028
**Lung**	0.511 ± 0.022	0.625 ± 0.188	0.539 ± 0.042	0.555 ± 0.024
**Kidney**	0.710 ± 0.025	0.720 ± 0.023	0.742 ± 0.061	0.715 ± 0.025
**Brain**	0.826 ± 0.040	0.770 ± 0.055	0.811 ± 0.072	0.895 ± 0.081
**Stomach**	0.038 ± 0.007	0.035 ± 0.007	0.042 ± 0.006	0.042 ± 0.013
**Adrenal gland**	0.255 ± 0.163	0.231 ± 0.068	0.301 ± 0.079	0.276 ± 0.040
**Thymus**	0.220 ± 0.313	0.080 ± 0.010	0.070 ± 0.010	0.075 ± 0.010
**Testis**	0.396 ± 0.373	0.245 ± 0.054	0.228 ± 0.033	0.251 ± 0.188

All values are expressed as the means (M) ± standard deviation (SD). ^*^p < 0.05, ^**^p < 0.01, ^***^p < 0.001. Values are compared with those of the control group. Differences were analysed by a one-way analysis of variance.

**Table 14:** Plasma arsenic species content in rats after oral administration of realgar for 30 days (*n* = 10, 5 males and 5 females per group, $\bar{x}$ ± SD).

Item	Control	Realgar		
		10.6 mg/kg	40.5 mg/kg	170 mg/kg
**As(III)**	0.02 ± 0.02	0.02 ± 0.02	0.02 ± 0.02	0.02 ± 0.01
**DMA**	7.91 ± 0.70	67.11 ± 2.29^***^	168.94 ± 8.92^***^	236.27 ± 8.05^***^
**MMA**	0.02 ± 0.01	0.04 ± 0.01	0.02 ± 0.02	0.04 ± 0.03
**As(V)**	0.08 ± 0.05	0.04 ± 0.03	0.04 ± 0.03	0.03 ± 0.02
**tAs**	8.49 ± 0.58	68.62 ± 2.66^***^	174.56 ± 10.98^***^	253.50 ± 23.33^***^

All values are expressed as the means (M) ± standard deviation (SD). ^*^p < 0.05, ^**^p < 0.01, ^***^p < 0.001. Values are compared with those of the control group. Differences were analysed by a one-way analysis of variance.

**Table 15:** Liver arsenic species content in rats after oral administration of realgar for 30 days (*n* = 10, 5 males and 5 females per group, $\bar{x}$ ± SD).

Item	Control	Realgar		
		10.6 mg/kg	40.5 mg/kg	170 mg/kg
**AsC**	0.004 ± 0.002	0.005 ± 0.003	0.005 ± 0.002	0.004 ± 0.003
**AsB**	0.003 ± 0.002	0.003 ± 0.002	0.062 ± 0.052	0.236 ± 0.128^*^
**As(III)**	0.009 ± 0.007	0.026 ± 0.025	0.074 ± 0.048	0.359 ± 0.122^**^
**DMA**	0.959 ± 0.145	10.321 ± 2.460^**^	23.099 ± 3.384^***^	31.678 ± 5.544^***^
**MMA**	0.018 ± 0.012	0.019 ± 0.013	0.019 ± 0.007	0.020 ± 0.014
**As(V)**	0.023 ± 0.01	0.029 ± 0.014	0.024 ± 0.012	0.021 ± 0.011
**tAs**	2.298 ± 0.379	15.545 ± 2.518^**^	28.135 ± 4.833^***^	38.300 ± 7.788^***^

All values are expressed as the means (M) ± standard deviation (SD). ^*^p < 0.05, ^**^p < 0.01, ^***^p < 0.001. Values are compared with those of the control group. Differences were analysed by a one-way analysis of variance.

**Table 16:** Kidney arsenic species content in rats after oral administration of realgar for 30 days (*n* = 10, 5 males and 5 females per group, $\bar{x}$ ± SD).

Item	Control	Realgar		
		10.6 mg/kg	40.5 mg/kg	170 mg/kg
**AsC**	0.002 ± 0.001	0.001 ± 0.001	0.001 ± 0.000	0.005 ± 0.007
**AsB**	0.003 ± 0.001	0.003 ± 0.001	0.002 ± 0.000	0.006 ± 0.009
**As(III)**	0.047 ± 0.006	0.055 ± 0.008	0.066 ± 0.007^*^	0.250 ± 0.122^*^
**DMA**	0.595 ± 0.113	3.973 ± 0.750^***^	11.435 ± 0.635^***^	18.470 ± 3.007^**^
**MMA**	0.004 ± 0.004	0.121 ± 0.105^**^	0.987 ± 1.844^**^	7.626 ± 1.844^*^
**As(V)**	0.015 ± 0.011	0.011 ± 0.010	0.024 ± 0.015	0.441 ± 0.433
**tAs**	3.678 ± 0.558	10.054 ± 2.813^**^	25.025 ± 7.365^**^	54.434 ± 15.210^*^

All values are expressed as the means (M) ± standard deviation (SD). ^*^p <0.05, ^**^p < 0.01, ^***^p < 0.001. Values are compared with those of the control group. Differences were analysed by a one-way analysis of variance.

**Table 17:** Brain arsenic species content in rats after oral administration of realgar for 30 days (*n* = 10, 5 males and 5 females per group, $\bar{x}$ ± SD).

Item	Control	Realgar		
		10.6 mg/kg	40.5 mg/kg	170 mg/kg
**AsC**	0.001 ± 0.000	0.001 ± 0.001	0.001 ± 0.000	0.002 ± 0.001
**AsB**	0.003 ± 0.001	0.004 ± 0.001	0.002 ± 0.000	0.003 ± 0.003
**As(III)**	0.041 ± 0.006	0.059 ± 0.008^*^	0.067 ± 0.009^**^	0.233 ± 0.073^*^
**DMA**	0.08 ± 0.05	0.845 ± 0.153^**^	2.166 ± 0.222^***^	2.997 ± 0.347^***^
**MMA**	0.02 ± 0.01	0.02 ± 0.01	0.03 ± 0.02	0.02 ± 0.01
**As(V)**	0.01 ± 0.01	0.02 ± 0.01	0.02 ± 0.01	0.02 ± 0.01
**tAs**	0.91 ± 0.18	1.80 ± 0.52	4.03 ± 1.07	5.70 ± 0.64

All values are expressed as the means (M) ± standard deviation (SD). ^*^p < 0.05, ^**^p < 0.01, ^***^p < 0.001. Values are compared with those of the control group. Differences were analysed by a one-way analysis of variance.

## 2 Experimental design, materials and methods

### 2.1 Single administration of realgar and biological sample collection

The rats were fasted for 16 h and then given a single realgar dose of 0 mg/kg (control) or 170 mg/kg (the realgar groups). Then, a blood sample was taken from the medial canthus vein of each mouse at 0.25, 0.5, 1, 2, 4, 8, 16, 32, and 48 h. Blood samples were anticoagulated and centrifuged to obtain plasma samples. Finally, rats were euthanized and their livers, kidneys and brains were removed.

### 2.2 Detection of the toxicity of realgar in rats after 30 days of oral administration [2], [3]

In total, 40 rats were randomly and equally divided into 4 groups as follows: ① the control group; ② the realgar 10.6 mg/kg/d group; ③ the realgar 40.5 mg/kg/d group; and ④ the realgar 170 mg/kg/d group. All rats were orally administered either 0.3% carboxymethylcellulose sodium (CMC-Na) in ultra-pure water (control group) or realgar (realgar groups) once daily for 30 consecutive days. At 31 days, the rats were anaesthetized using an intraperitoneal injection of phenobarbital sodium. Blood samples were then collected from the abdominal aorta for haematology, coagulation, biochemical and electrolyte analyses. Finally, the rats were sacrificed, and all organs were excised, observed, and weighed.

### 2.3 Analysis of the accumulation and distribution of As species in biological samples [4]

An Agilent 7700ce ICP-MS instrument coupled with the Agilent 1200 Series HPLC system was used for quantitative analyses of the tAs and As species content in rats plasma and organs.
